# The Antioxidant Mechanism of Peptides Extracted from Tuna Protein Revealed Using a Molecular Docking Simulation

**DOI:** 10.3390/antiox13020166

**Published:** 2024-01-29

**Authors:** Xiaojun Guo, Jiaxin Liu, Cheng Wang, Zhengshun Wen, Bin Zheng

**Affiliations:** 1 School of Food Science and Pharmaceutics, Zhejiang Ocean University, Zhoushan 316022, China; guoxiaojun@zjou.edu.cn (X.G.); liujiaxin@zjou.edu.cn (J.L.); 2 Xianghu Laboratory, Hangzhou 311231, China

**Keywords:** tuna protein, antioxidant activity, activity peptides, molecular docking

## Abstract

Tuna protein serves as a significant source of bioactive peptides, and its functional properties can be elucidated through predictive modeling, followed by experimental validation. In this study, the active polypeptides were obtained from tuna protein via enzymatic hydrolysis (TPP), and their peptide sequences were determined. Furthermore, the potential activity of these peptides was predicted, focusing on antioxidant peptides, and compared to the sequence library of known antioxidant peptides to identify common structural motifs. The accuracy of the prediction results was confirmed through in vitro antioxidant assays and molecular docking studies. We identified seven specific peptide segments derived from tuna protein that exhibit antioxidant potential, accounting for approximately 15% of all active peptides. Molecular docking and cell experiments were employed to provide compelling evidence for the presence of antioxidant peptides within tuna protein. This study not only lays a solid foundation for studying the structure of active peptides but also opens up a novel avenue for an expedited assessment of their properties.

## 1. Introduction

Tuna red meat is characterized by its high protein content of up to 20% and low lipid count; its amino acid composition aligns with human nutritional needs, marking it as a high-quality protein source, rich in unsaturated fatty acids, and it stands as a pivotal subject for scientific exploration [1]. Despite the widespread availability of tuna products like canned fish and sashimi on the market, the production processes themselves result in significant quantities of processing by-products, such as fish skin, offal, and bones. These by-products are typically categorized as low-value or waste materials, which are either utilized for bait or animal feed, or simply discarded. This is primarily because the demand for these processed by-products is considerably limited on the commercial market [2]. However, a further examination reveals a significant protein content suitable for the extraction of potent active peptides via enzymatic hydrolysis and fermentation. As a result, tuna appears not only as a primer source of protein, but also garners attention from economic and environmental standpoint due to its potential for active peptide extraction [3]. We enzymatically hydrolyzed tuna red meat flesh using the enzymatic hydrolysis method [4]. After separation and purification, the resulting protein hydrolysate provided active peptides with scrutinized antioxidant characteristics that were investigated. Antioxidant peptides primarily block oxidation reactions in three ways [5]: (1) scavenging free radicals by providing hydrogen atoms or electrons through peptide bonds and hydroxyl groups of antioxidant peptides, so as to eliminate the destruction of free radicals to biomolecules; (2) these compounds block the progression of oxidation reactions by inhibiting oxidase activity, chelating metal ions that catalyze oxidation reactions, and creating a protective layer around lipid particles; (3) it increases the level of antioxidants such as glutathione (GSH), catalase, and superoxide dismutase [6], acting as a regulatory role in cellular physiology, and collaborating with antioxidant enzymes to prevent the onset of chronic diseases caused by organelle destruction [7]. Peptides produced from tuna have been shown to exhibit antioxidant activity in numerous studies, demonstrating their ability to scavenge free radicals, reduce potential, and modulate enzymes connected to antioxidants.

Molecular docking is a crucial technique used in computer-aided drug research and plays a significant role in understanding and investigating the interactions between enzymes and substrates. Molecular docking offers a remarkable approach to uncover the intricate relationship between the active site of a protease and its substrate or inhibitor, drawing a parallel between this relationship and the mechanism of a key and lock. The concept essentially suggests that just like a key need to fit perfectly into a lock, the ligand (substrate or inhibitor) must undergo complementary changes upon contact with the receptor in order to achieve compatibility. These alterations occur simultaneously in both the ligand and receptor, allowing them to bind effectively and form a stable complex. Through molecular docking, scientists can gain insights into the structural and functional aspects of the ligand–receptor interaction, aiding in the discovery and design of potential drugs with higher specificity and efficacy. The second aspect is matching, in terms of energy, in which the free energy will change and affect the strength of their bond [8]. Molecular docking is a common method, which allows direct observation of interaction between small molecule peptides and biological targets [9].

At present, the most common procedure for obtaining active peptides is to prepare them by processing, separating, and purifying proteins. Among the various methods available for this purpose, enzymatic hydrolysis has gained popularity due to its numerous advantages. This method is preferred because it operates under mild conditions, making it suitable for preserving the delicate structure and functionality of polypeptides. Additionally, enzymatic hydrolysis is cost-effective and safe, making it a desirable choice for large-scale production. Furthermore, the use of different enzymes, either alone or in combination, can lead to varied results, enabling researchers to obtain a diverse range of polypeptides with distinct properties. However, it is important to note that enzymatic hydrolysis typically generates a mixture of peptides, rather than a single, pure polypeptide, to obtain a single peptide segment and study its functional activity, for which a significant amount of separation work is required [10]. However, the traditional methods for assessing the functional activity of active peptides are tedious and require a lot of time. This creates a need for new and innovative methods to evaluate the activity of these peptides. In order to address this need, our study used high-throughput screening based on mass spectrometry to quickly analyze tuna hydrolysate. We then used activity prediction and molecular docking visualization to identify the main active peptides in the hydrolysate. To validate the activity of peptides, we measured their ability to scavenge free radicals and regulate cellular antioxidant enzymes. This comprehensive approach not only provides a solid foundation for studying the structure of active peptides, but also presents a novel avenue for the expedited assessment of active peptide properties.

## 2. Materials and Methods

### 2.1. Reagents

Tuna, Zhejiang Xingye Group (Zhoushan, China); DPPH free radical scavenging ability kit, total superoxide dismutase (SOD) assay kit, hydrogen peroxide (CAT) assay kit, glutathione peroxidase (GSH-PX) assay kit, malondialdehyde (MDA) assay kit (Nanjing Jiancheng Co., Nanjing, China); ethanol, ferrous sulfate, salicylic acid, 30% H_2_O_2_, Tris, pyrogallol.

### 2.2. Preparation of Polypeptide from Tuna

Bioactive peptides were extracted from tuna protein. Frozen tuna was thawed out in a washing tank with flowing water for 2 h before being cleaned with clean pure water. The ratio of red meat raw materials to pure water liquid is 1:1.5 g/mL, cook at 95 °C for 0.5 h, stir while heating; after steaming, cooled to 55 °C. First, adjust the pH to 9 with 1 mol/L NaOH, and then add alkaline protease according to 1% of the quality of raw materials. Stir enzymatic hydrolysis solution for 1 h, and then adjust the pH to 7 with 1 g/L citric acid. Add complex protease according to raw material 2%, and stir enzymatic hydrolysis for 2 h. Following the completion of the enzymatic hydrolysis, the temperature was raised to 85 °C, agitated for 30 min, and centrifuged and filtered at 3000 rpm/min with a centrifuge. It was decolorized with activated carbon after filtration, and activated carbon was added in the proportion of 4% of the raw material mass, heated to 70 °C, and sonicated and stirred for 30 min. After the reaction was completed, the mixture underwent centrifugation and filtration at 3000 rpm/min, and then the sample was concentrated and steamed while the temperature was kept at 55 °C until the solid content of the enzymatic hydrolysate was around 20%. Finally, the enzymolysis solution was sprayed with a spray dryer set at 150 °C with a flow rate of 100 mL/h to obtain a polypeptide from tuna (TPP).

### 2.3. Peptide Sequence Identification with Q-TOF MS/MS

Using TPP as raw material, the enzymatic hydrolysis solution was obtained with enzymatic hydrolysis. After the enzymatic hydrolysis solution was centrifuged at high speed, 40 μL of the supernatant was taken, diluted 40 times with water, and 1 μL was drawn and spotted on the target plate of the 5800 Q-TOF MS/MS instrument. Q-TOF MS/MS was used for sequence analysis of the enzymatic hydrolysate after injection. The mass spectrum of the sample obtained with this analysis was collected and compared with the spectral library using ProteinLunxGlobal Server 3.0.3 to confirm the LC-MS/MS mass spectrometry analysis of the polypeptide’s primary structure.

### 2.4. Overall Activity Prediction of TPP

PeptideRanker was used to predict the activity of TPP (http://distilldeep.ucd.ie/PeptideRanker/ (accessed on 23 March 2023)), a server for predicting biologically active peptides based on novel neural networks. The probability that the peptide is biologically active is returned to the user, with a threshold of 0.5, and any peptide predicted to exceed the 0.5 threshold is flagged as biologically active. PeptideRanker makes predictions regarding amino acid sequences and does not involve peptide modifications.

### 2.5. Antioxidative Activity and Toxicity Prediction of Effective Peptides

Antioxidant activity prediction was used for biological activity predictions over 0.5 (https://services.healthtech.dtu.dk/services/AnOxPePred-1.0/ (accessed on 25 March 2023)). The threshold was 0.5, and any peptide predicted to exceed the 0.5 threshold was flagged as having antioxidant activity. Toxinpred (http://crdd.osdd.net/raghava/toxinpred/ (accessed on 25 March 2023)) program was used to predict the toxicity of the selected peptides.

### 2.6. Antioxidant Active Peptide Comparison

This study involved a comprehensive summary and analysis of the antioxidant peptides present in a protein database. Specifically, we focused on examining the distribution of amino acids at the C-terminal and N-terminal ends of these antioxidant peptides, and the peptide sequences predicted to have antioxidant activity were compared.

### 2.7. Validation of Antioxidant Activity

The activity of the peptide derived from tuna can be verified with chemical experiments to observe its 2,2-Diphenyl-1-picrylhydrazyl (DPPH), hydroxyl radical, and superoxide anion clearance.

#### 2.7.1. Determination of DPPH Free Radical Scavenging Rate

The determination method of DPPH clearance is improved based on Liu et al. [11]. Sample solutions of different concentrations and 0.1 mmol/L DPPH ethanol solutions were prepared. Accurately pipette 0.4 mL of the sample to be tested into a 5 mL centrifuge tube, add 0.6 mL of DPPH ethanol solution, mix well, place in the dark for 30 min, shake continuously, and measure the absorbance at 517 nm. DPPH clearance rate (%) = ((A_1_ − (A_2_ − A_3_))/A_1_) × 100, where A_1_ is 0.4 mL of 80% methanol solution + 0.6 mL of DPPH solution; A_3_ is 0.4 mL of sample + 0.6 mL of 80% methanol solution; A_2_ is 0.4 mL sample + 0.6 mL DPPH solution. Trolox was selected as the positive control. Record the concentration of DPPH free radical scavenging rate of the sample to be tested when it reaches 50%, which was called the half-maximal inhibitory concentration (IC_50_), This represents the effective concentration of the polypeptide molecule required to remove 50% free radical activity.

#### 2.7.2. Determination of Hydroxyl Radical Scavenging Rate

On the basis of Cao et al. [12], the determination method of hydroxyl radical scavenging effect was improved. Sample solutions of different concentrations—9 mmol/L FeSO_4_ solution, 9 mmol/L salicylic acid–ethanol solution, and 8.8 mmol/L H_2_O_2_ solution—were prepared. Accurately pipette 0.4 mL of the sample to be tested into a 5 mL centrifuge tube, add 0.4 mL of salicylic acid–ethanol solution, 0.4 mL of FeSO_4_ solution, and 0.4 mL of H_2_O_2_ solution, mix well, and measure the absorbance at 510 nm. Hydroxyl radical scavenging rate (%) = ((A_0_ − (A_1_ − A_2_))/A_0_) × 100, where A_0_ is 0.4 mL of water + 0.4 mL of salicylic acid–ethanol solution + 0.4 mL of FeSO_4_ solution + 0.4 mL of H_2_O_2_ solution; A_1_ is 0.4 mL sample + 0.4 mL salicylic acid–ethanol solution + 0.4 mL FeSO_4_ solution + 0.4 mL H_2_O_2_ solution; A_2_ is 0.4 mL sample + 0.4 mL salicylic acid–ethanol solution + 0.4 mL FeSO_4_ solution + 0.4 mL distilled water. Vitamin C (Vc) was selected as the positive control, and the IC_50_ of the sample concentration to be tested was calculated when the hydroxyl radical scavenging rate reached 50%, indicating the effective concentration of polypeptide molecules required for scavenging 50% of the free radical activity.

#### 2.7.3. Determination of Superoxide Anion Scavenging Rate

The activity of superoxide anion scavenging was slightly modified from the previous method of Misak et al. [13]. Sample solutions of different concentrations, 50 mmol/L Tris-HCl buffer solution, 3 mmol/L pyrogallol solution, and 10 mmol/L HCl solution were prepared. Accurately pipet 1 mL of the sample to be tested into a 10 mL centrifuge tube, add 5 mL of Tris-HCl buffer solution, react at 25 °C for 10 min, add 1 mL of pyrogallol solution, stop the reaction with 1 mL of HCl solution, and measure the absorbance at 320 nm. Superoxide anion clearance rate (%) = ((A_0_ − (A_1_ − A_2_))/A_0_) × 100, where A_0_ is 1 mL of water + 5 mL of Tris-HCl buffer solution + 1 mL of pyrogallol solution + 1 mL HCl solution; A_1_ is 1 mL sample + 5 mL Tris-HCl buffer solution + 1 mL pyrogallol solution + 1 mL HCl solution; A_2_ is 1 mL sample + 5 mL Tris-HCl buffer solution + 1 mL water + 1 mL HCl solution. Vc was selected as the positive control, and the IC_50_ of the sample concentration to be tested was calculated when the superoxide anion scavenging rate reached 50%, indicating the effective concentration of polypeptide molecules required to scavenge 50% of free radical activity.

### 2.8. Cell Culture and Antioxidant Test

Dulbecco’s modified eagle medium (DMEM) complete culture solution was used to cultivate human colorectal adenocarcinoma cells (LS174T). The solution contained 10% fetal bovine serum by volume and 1% antibiotic (100 U/mL penicillin, 0.25 µg/mL streptomycin) in 5% CO_2_ cell culture at 37 °C. For the experiment, LS174T cells in the exponential growth stage were employed.

After being removed, the LS174T cells were injected on the well plate for 24 h at a logarithmic growth stage. Following the end of the culture, samples from the concentration gradient were added to the culture medium and were then replaced with 1000 μmol/L H_2_O_2_ for 12 h. Establish the normal group (CON), 1000 µmol/L H_2_O_2_ module (MOD), 200 µg/mL of low-dose group peptide (TPL), 600 µg/mL dose peptide group (TPM), and 800 µg/mL high dose group peptide (TPH). The contents of SOD, CAT, GSH-Px, and MDA in the cell lysate were determined by the kit.

### 2.9. Molecular Docking

Molecular docking method was used to study the inhibitory mechanism of tuna polypeptide with DPPH, hydroxyl radical, superoxide anion, and oxidase related pathway protein Keap1. Ligand DPPH (CID: 2735032), hydroxyl radical (CID: 2735032), and superoxide anion (CID: 5359597). Three-dimensional structure of downloaded from PubChem database (https://pubchem.ncbi.nlm.nih.gov/ (accessed on 15 May 2023)). Three-dimensional structure of Keap1 (PDB: 7Q6S) comes from bank PDB database (https://www.rcsb.org/ (accessed on 15 May 2023)).

First, after predicting all the peptides, the one with the highest predicted antioxidant activity score was carefully selected. To further analyze its structure, the 2D structure of the peptide was constructed using ChemDraw Professional 16.0 software. Subsequently, the 3D structure of the peptide was generated by converting the 2D structure using Chem3D 16.0. To ensure the most favorable energy state, optimization was performed on the 3D structure.

For the molecular docking process, the peptide was used as a receptor while DPPH, hydroxyl radical, and superoxide anion served as ligands. To encompass all the necessary molecules, the active bag size of the peptide was configured. By using molecular docking techniques, the peptide was effectively docked with each of the ligands separately. Moreover, the peptide was also subjected to molecular docking with the Keap1 protein. In this case, the peptide acted as the ligand while the Keap1 protein functioned as the receptor. Prior to docking, excess water and polyconformation proteins were removed from the Keap1 protein using PyMOL 2.3.4 software. Furthermore, Autodock 1.5.7 was used to optimize the receptor by performing hydrotreatment. Consequently, an active bag containing both peptides and proteins was established and subjected to docking. To enhance the accuracy of the docking results, the conformation of the peptide and ligand interactions was optimized using a genetic algorithm based on the minimum docking energy principle. This step aimed to find the most favorable spatial arrangement and bonding between the peptide and ligands, with an analysis of both the stereoscopic and planar arrangements.

### 2.10. Statistical Analyses

In this study, the results were showed as mean ± standard deviation (SD). All data were subjected to one-way analysis of variance (ANOVA), and Duncan’s method was used to test significant differences among trial groups using SPSS 23.0 (SPSS Inc., Chicago, IL, USA). *p* < 0.05 was considered statistically significant. Correlations were obtained using Pearson’s correlation coefficients at the same level of significance. Violin plots were performed using Origin 2021.

## 3. Results and Discussion

### 3.1. Results of Enzymatic Hydrolysis of TPP

According to Section 2.2, 2000 g of tuna protein was enzymolized, and 214 g of TPP was obtained by spray drying the enzymolysis solution, with a yield of 10.7%.

### 3.2. Atlas Characterization Analysis of TPP

The Q-TOF MS/MS analysis revealed two prominent mass spectral peaks, with ion *m*/*z* 92.95 having the highest signal intensity, followed by *m*/*z* 80.25. Several other signals also exhibited substantial strength. Interpretation of the mass spectrometry outcomes involved peptide sequence analysis conducted through PLGS (ProteinLynx Global Server). This system utilizes the Apex3D algorithm to combine isotopes and charge states from a single peptide or fragment into a single result, expressed as MH + *m*/*z*, average RT (and drift time), and the total intensity of all charges and isotopes. Finally, it compares the results with an existing sequence library, and the peptide fragments are matched to peptides with the same drift time and peak shape.

The peptides were matched, and 293 peptides were identified in TPP, which were matched with the tuna protein sequence library. The corresponding protein sequence was found to be as follows: A4L7J0, Q9IB35, Q6I7B0, I0J1J1, G9M5T2, G9M5T1, D4HTS6, Q9IB36, Q9IB37, Q9IB34, A0A292G2Z7, A0A060N2D3, Q76CT4, A0A1C9U5W5, Q8AYM0, M4QB37, U3U9M8, Q6S9V9, A0A1D0A1I9, P11749, A9QKS0.

### 3.3. Prediction of Overall Peptides Activity and Toxicity

The peptide spectrum analysis, which involved examining a set of 293 peptides, aimed to determine their biological activity. In order to assess their activity, a threshold value of 0.5 was employed, whereby any predicted value greater than 0.5 was categorized as an active peptide. The analysis results, outlined in Table 1, revealed that a total of 54 peptides displayed noticeable activity after the prediction process.

Here, the predicted results comprise the overall activity of peptides, with the functions of bioactive peptides being diverse. With an increasing body of evidence, some peptides extracted from animals have been found to inhibit the growth of Gram-positive bacteria, proving that they have antibacterial activity [14]. The most studied bioactive peptides are antioxidant peptides. Moreover, oxidative stress is closely related to various chronic diseases, and most of the peptides can play a positive role by inhibiting oxidative stress channels and affecting antioxidant enzymes.

### 3.4. Prediction of Antioxidant Activity of Overall Functionally Active Peptides

Initially, the activity of the peptides was anticipated, and the antioxidant activity of 54 active peptides was predicted. The prediction was based on amino acid composition and SVM score analysis. The following physical and chemical parameters were assessed: hydrophobicity, hydrophilicity, amphiphilicity, steric hindrance, and molecular weight. Peptides having an SVM score of 0.5 or above were judged to have antioxidant activity potential. The results are presented in Table 2. There are seven peptides that had antioxidant activity potential, making up 15% of all active peptides. Subsequently, the toxicity prediction of the seven peptides in the data revealed no toxicity, allowing a follow-up antioxidant activity study to be carried out.

### 3.5. Structural Comparison of Antioxidant Active Peptides

**Figure 1:**
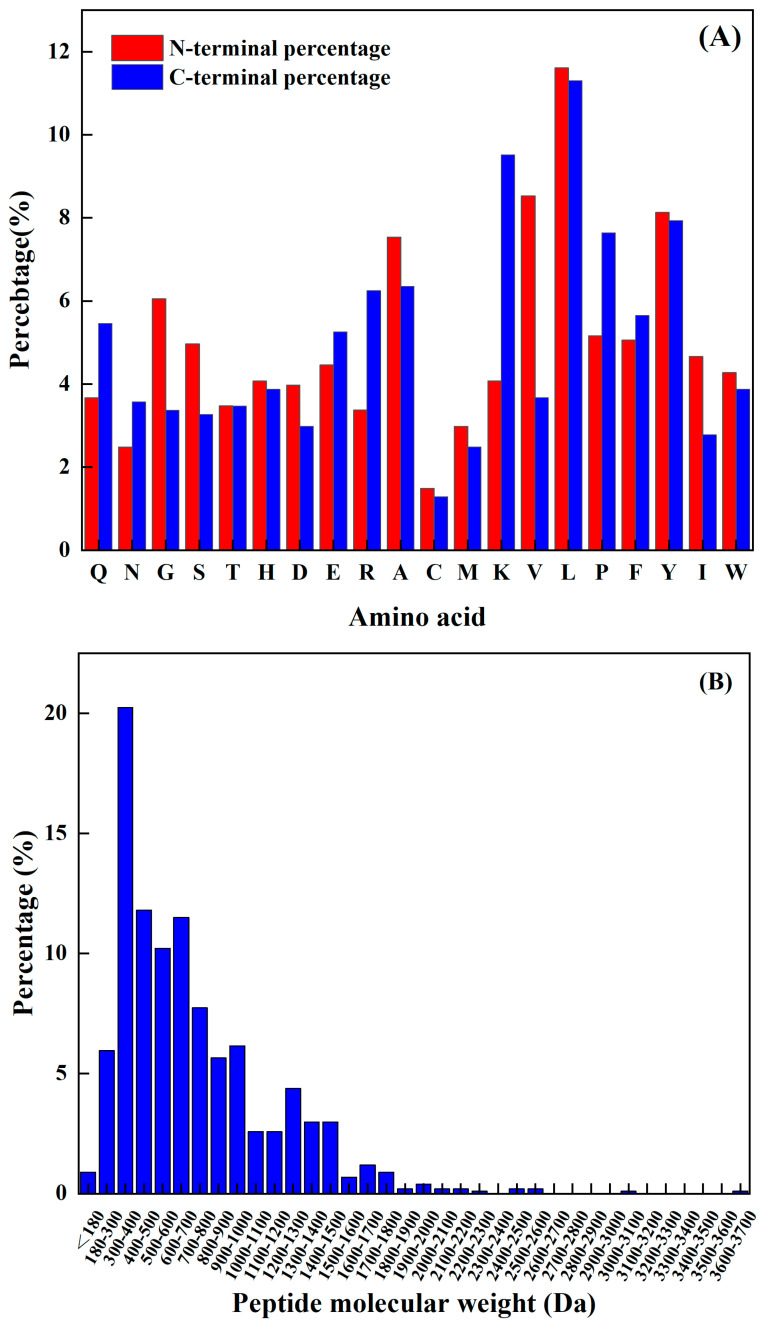
(**A**) Frequency of different amino acids at the N–terminal and C–terminal, and (**B**) antioxidant active peptide molecular quantity distribution.

The amino acids at the N-terminal and C-terminal of the peptide have a significant impact on its functional activity, and not only improve their functionality but may also be vital spots for scavenging free radicals. Sequence analysis, shown in Figure 1A, reveals that the N-terminus has a relatively high frequency of L, K, Y, P, and A, while the C-terminus has a comparatively high frequency of L, V, Y, A, and G. The predicted seven peptides’ N-terminal and C-terminal amino acids all have a high frequency; hence, the prediction findings are very accurate.

The molecular weight of a polypeptide has a great impact on its oxidation characteristics. According to the research findings, when analyzing the composition of antioxidant peptides, it was observed that these peptides primarily consist of a maximum of 20 amino acids. Additionally, it was discovered that the antioxidant peptides with lower molecular weights have a smaller spatial profile. This characteristic is advantageous as it allows these peptides to establish a closer proximity to free radicals. Consequently, the smaller spatial profile of lower-molecular-weight antioxidant peptides enables them to directly interact with free radicals more efficiently. Kim et al. [15] found that the molecular weight of the peptide was an important factor affecting its antioxidant properties. The cod protein hydrolysis product component of the low-molecular-weight component (1–3 kDa) had a higher antioxidant capacity compared to the other polymer’s active quantity components. Related reports are common. For example, Ranathunga et al. [16] studied peptides from Congieel and found that the low-molecular-weight group showcased superior antioxidant capabilities in comparison to natural antioxidants.

The analysis of the existing 1008 peptide sequences in the protein sequence library as carried out. As shown in Figure 1B, the molecular weight of the known antioxidant activity was found to be less than 3.7 kDa, and was more distributed from 180 Da to 1900 Da. Table 2 shows antioxidant peptides and their corresponding molecular weights. It was found that four peptides are in the range from 1200 Da to 1900 Da, and the other four are less than 1000 Da.

### 3.6. Chemical Verification of Antioxidant Activity of Peptides

**Figure 2:**
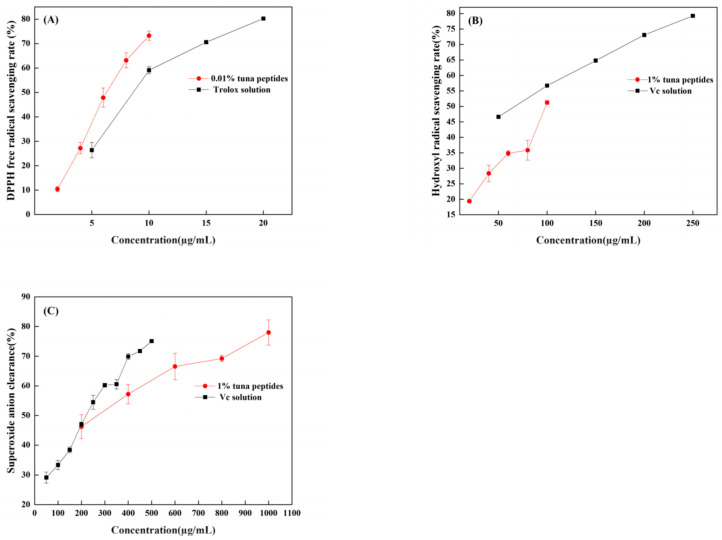
(**A**) DPPH radical scavenging capacity of tuna polypeptide and Trolox, (**B**) hydroxyl radical scavenging capacity of golden tuna polypeptide and Vc, and (**C**) superoxide anion scavenging capacity of tuna polypeptide and Vc (*p* < 0.05).

When DPPH is dissolved in ethanol, it turns a dark purple color. The electrons of lone pairs are paired with each other under the impact of antioxidants; thus, the dark purple gradually lightens and the light absorption ability weakens. The strength of the light absorption value can be used to assess antioxidant scavenging activity. As shown in Figure 2A, both TPP and Trolox can scavenge DPPH radicals in a dose-dependent manner, with IC_50_ values of 66.96 mg/mL and 11.23 µg/mL, respectively. In a certain concentration range, the scavenging effect of tuna peptides and Trolox on the DPPH free radical was linear with a change in sample concentration. The scavenging effect of Trolox on the DPPH free radical was significantly higher than that of the tuna peptides. Because of the presence of hydrophobic amino acids with lower molecular weights in peptides, they have an enhanced penetration target organ via hydrophobic contact. Lapsonfon et al. [17] pointed out that sulfur-containing amino acids, including cysteine, can provide hydrogen on mercaptan groups, improving the scavenging ability DPPH radicals. Therefore, we consider that TPP, as a mixed peptide, has a higher overall molecular weight as a whole, so its DPPH scavenging ability is poor.

The efficiency of natural antioxidants in neutralizing hydroxyl free radicals serves as an indicator of their activity level and their potential antioxidant function. As depicted in Figure 2B, there was a substantial increase in the clearance rate with the escalation of tuna peptides and Vc. The IC_50_ values were 10.5 mg/mL and 63.66 µg/mL, respectively, indicating the robust hydroxyl radical scavenging ability of tuna peptides.

The superoxide anion radical has significant physiological effects and is closely associated with various diseases, it is the primary free radical in the oxygen free radical, it is a very potent oxidation, and it has the ability to produce a series of oxidation reactions to generate other oxygen free radicals. Thus, its removal is important. Figure 2C indicates that tuna peptides and Vc can be oxidized using superoxide anions. The higher the concentration of the sample, the greater the free radical scavenging ability of the tuna peptide and Vc. The IC_50_ values were 24.40 mg/mL and 238.77 µg/mL. Niranjan et al. [18] found that a higher concentration of protein peptides enhances the scavenging capability of superoxide anions.

Enzyme hydrolysis produced tuna peptides exhibiting antioxidant activity. This was validated through assessments of DPPH, hydroxyl, and superoxide anion scavenging capabilities. This study conducted analyses and discussions on the structure of peptides, specifically focusing on the role of the amino acid Tyr in relation to their antioxidant properties. Through these analyses, it was revealed that Tyr plays a crucial role in terminating the chain reaction of lipids, which is important for protecting cells from oxidative damage. This amino acid also exhibited a remarkable ability to scavenge free radicals, thereby preventing their harmful effects to be elucidated. The results obtained from this study demonstrated that, among the three indexes tested (superoxide anion, hydroxyl radical, and DPPH), the scavenging rate of the superoxide anion radical was the highest, followed by hydroxyl radicals, with DPPH exhibiting the lowest scavenging ability. These findings suggest that the superior antioxidant potential observed in the predicted peptide AGLYPGA could be attributed to its high content of Tyr. Wu et al. [19] determined the free radical scavenging ability of free amino acids via oxygen free radical absorbance and found that the free radical scavenging ability order was as follows: Trp > Tyr > Met > Cys > His. Moreover, it was found that these amino acids all contribute to the antioxidant activity of peptides.

### 3.7. Effects of Active Peptides on Cellular Antioxidant Enzymes

**Figure 3:**
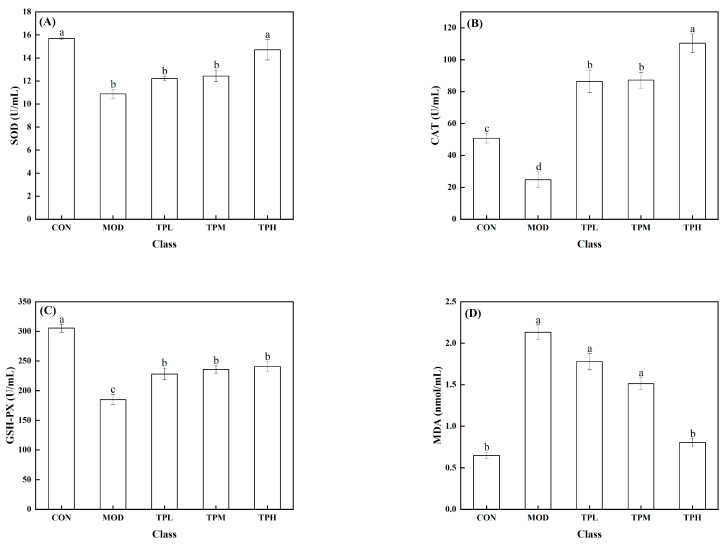
Effect of TPP on LS174T cell oxidation-associated enzymes. (**A**) SOD, (**B**) CAT, (**C**) GSH-Px, and (**D**) MDA. There was a significant difference in the activity of various enzymes in the data marked with different letters (*p* < 0.05).

As shown in Figure 3, a significant decrease in the activities of SOD, CAT, and GSH-PX was found in the model group exposed to H_2_O_2_ compared to the control group, along with a large increase in the MDA level. SOD, CAT, and GSH-Px are intracellular antioxidant enzymes that can prevent free radicals from damaging cell membranes and maintain cell health. The increase in the MDA level has an effect on cell survival. MDA levels that are too high can interfere with proper cell viability. Different dosages of tuna polypeptides were administered, resulting in different effects. The results showed that a high polypeptide dose had an effect on the activity of the SOD enzyme, whereas the low and medium doses had no discernible effect. Low and medium doses of tuna peptides can impact the levels of CAT and MDA, whereas high doses of tuna peptides can produce more obvious effects. The enzyme activity of GSH-PX was affected by low, medium, and high doses, but there was little variation between them. These findings show the tuna polypeptide’s potential for efficiently regulating antioxidant enzyme activity, with higher doses demonstrating more pronounced regulatory effects.

### 3.8. Optimal Docking Model of Peptides and DPPH, Hydroxyl Radical, Superoxide Anion, and Keap1

The molecular docking technique can demonstrate the interaction between antioxidant peptides and free radicals more clearly, and is a technology with significant advantages and prospective benefits for antioxidant drugs and other fields of research [20]. The affinity data were obtained by simulating the docking of the peptide sequence AGLYPGA with DPPH, hydroxyl radical, and superoxide anion molecules using AutoDock Vina software. This is an important method for determining the best ligand. The binding affinity between the receptor and ligand is influenced by serval factors, including the spatial effect, repulsive force, hydrogen bond, and hydrophobic interaction. The docking score usually comprises negative values, with a higher absolute value indicating a more beneficial docking effect [21].

**Figure 4:**
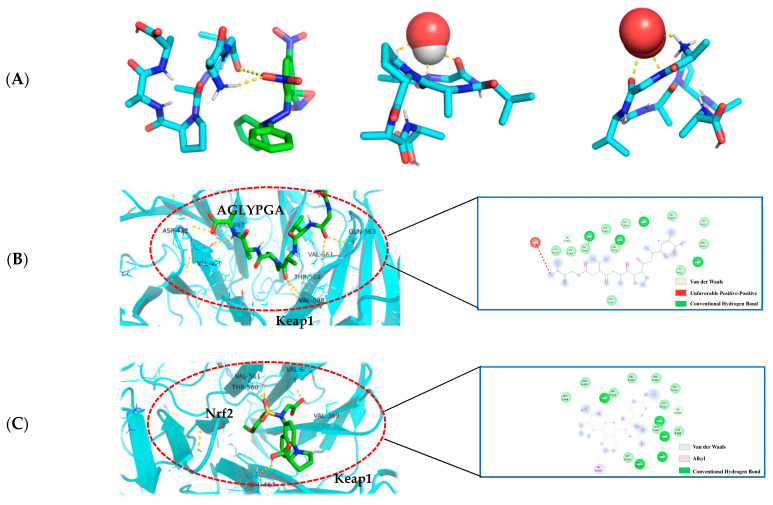
(**A**) AGLYPGA is docked with DPPH, hydroxyl radicals, and superoxide anions, respectively; (**B**) 3D and 2D diagram of AGLYPGA docking with Keap1; and (**C**) Keap1-Nrf2 coupling mode.

In this paper (as shown in Figure 4A), according to the minimum binding energy after docking, the optimal binding configuration of the peptide segment AGLYPGA and DPPH, hydroxyl radical, and superoxide anion with the best prediction result of antioxidant activity was obtained. The peptide formed two hydrogen bonds with DPPH, one hydrogen bond with the hydroxyl radical, and three hydrogen bonds with the superoxide anion. To compare, there are more hydrogen bonds between peptides and free radicals. This is related to Tyr’s ability to terminate the lipid chain reaction more optimally and due to it having a stronger free radical scavenging ability. Based on the above results, the peptide has a strong antioxidant capacity, which is consistent with the results of the in vitro antioxidant verification test.

The Keap1-Nrf_2_ signaling pathway is an important system for antioxidant function, but it can cause oxidative damage within the body when activated excessively [22]. When oxidative damage occurs, Keap1 interacts with free radicals, and peptides can change the Keap1/Nrf_2_-ARE signaling pathway, influencing the role of the antioxidant enzymes in cells [23]. Studies have shown that a peptide extracted from soft-shell turtles can enhance the activity of ARE by up-regulating Nrf_2_ and down-regulating Keap1. Wang et al. [24] performed molecular docking of the peptide with Keap1 and found that the peptide residues were bound to the active site of Keap1. In order to examine the antioxidant effect of tuna peptides, this study delves into the molecular docking analysis of the peptide AGLYPGA with Keap1 protein.

The antioxidant activity of epigallocatechin glycerate (EGCG) demonstrates a binding energy of −6.62 kJ/mol [25]. In comparison, the binding energy of AGLYPGA is −7.6 kJ/mol, demonstrating its stable binding capacity with Keap1 and potential steric hindrance impact. This steric hindrance eventually inhibits the Keap-Nrf_2_ interaction, increasing the production of antioxidant enzyme genes, which is consistent with the predicted results [26]. Moreover, it was demonstrated that peptides screened from tuna eggs contribute to the increased antioxidant levels by altering the Keap1/Nrf_2_-ARE pathway [27], which is compatible with the current findings. As shown in Figure 4C, the major amino acid residues to which Keap1-Nrf_2_ interacts with include VAL-467, VAL-561, ALA-607, THR-560, VAL-369, VAL-608, VAL-420, GLY-419, CYS-513, and IEU-468. In Figure 4B, the combination model of AGLYPGA and Keap1-Kelch region was compared with the Keap1-Nrf2 model, and many similarities were found. The main amino acid residues that it binds to comprise HIS-516, LEU-515, ALA-607, GLY-564, GIL-563, VAL-369, VAL-561, VAL-514, VAL-608, VAL-420, VAL-467, ASP-422, ILE-421, and THR-560. During the analysis, it was discovered that both models had precisely the same sequence of amino acid residues, including THR-560, VAL-467, VAL-561, ALA-607, VAL-369, VAL-608, and VAL-420. Compared to previous studies, the results of this study were similar to those of Li et al. [28], whose study involved a peptide extracted from eggs. Their research found that peptide segments’ amino acids can occupy the binding site of the Keap1-Kelch domain, thereby inhibiting the Keap1-Nrf_2_ interaction. Thus, the peptides derived from tuna can exert antioxidant effects by binding to Keap1.

## 4. Conclusions

The Q-TOF MS/MS technique was used to analyze the peptides generated from tuna. The antioxidant activity of these peptides was predicted by comparing them to an antioxidant protein sequence library. Following that, chemical verifications were performed to evaluate DPPH scavenging ability, hydroxyl radical scavenging ability, and superoxide anion scavenging ability, allowing for an assessment of overall antioxidant resistance. The binding between the peptide with the highest predicted score and Keap1 was visualized using molecular docking, confirming the prediction’s correctness. Our findings highlighted tuna polypeptides’ potent hydroxyl radical and superoxide anion scavenging activities, indicating their high overall antioxidant activity. Among the eight anticipated peptides (AGLYPGA, GEPGPAG, LPGGGPVL, APVAPGYGGG, AAAPAPAPAPAPAPA, AEPAPAPAPAPEPAPAPA, KAEPAPAPAPAPEPAPAPA, AEPAPAPAPAPEPAPAPAAPA), the peptide AGLYPGA with the greatest predicted score was verified and validated using molecular docking. This peptide had a high affinity for Keap1, implying that it has the potential to regulate the Keap1/Nrf2-ARE pathway, hence increasing the antioxidant levels. The study approach of functional peptide prediction followed by verification can be used as a reference for future research. The study strategy of first predicting functional peptides and then validating them can be a useful reference for future research endeavors in this field.

## Figures and Tables

**Table 1:** Predicted results of peptide activity.

Peptide Sequence	Molecular Weight	Activity Score	Peptide Sequence	Molecular Weight	Activity Score
FDTFLPM	869.3993	0.918121	AEPAPAPAPAPEPAPAPA	1620.8147	0.614793
PGGVPFLP	814.4225	0.895559	DLGGGTF	665.302	0.612586
PAPAPAPAAGGT	982.5059	0.844022	AGPPGSTGP	739.35	0.601717
ASGPINF	704.3493	0.842426	GPSGPAGPTA	739.35	0.596112
APAPAPAPAAG	911.4476	0.803473	AAGPPGSTGP	810.3871	0.593769
APVAPGYGGG	876.3977	0.802741	LPGGGPVL	707.433	0.586057
GPAGASGPAGPR	993.4991	0.782288	KAEPAPAPAPAPEPAPAPA	1748.9097	0.585361
APAPAPAPAPAPA	1168.624	0.767656	VILPVPAF	854.5266	0.582872
AGPSGPR	640.3292	0.765178	SKTPGLM	746.3997	0.582128
APAPAPAPAPA	929.497	0.762092	PFDQDDWE	1050.3931	0.579299
APAPAPAPA	761.4071	0.760005	HVDPDNF	842.3558	0.574675
AAPAPAPAPAPAPA	1168.624	0.75938	KKAEPAPAPAPAPEPAPAPA	1877.0046	0.570387
SGPINFT	734.3599	0.753769	AAIIKPL	724.4847	0.565087
ASGPINFT	805.397	0.751915	AEPAPAPAPAPEPAPAPAAPA	1859.9417	0.563469
APAPAPAAG	743.3452	0.748276	ADFDAVL	791.3701	0.556689
IDFGMDL	809.3629	0.746159	AKKAEPAPAPAPAPEPAPAP	1877.0046	0.555846
AAAPAPAPAPAPAPA	1239.6611	0.730839	GRGGAGGL	643.3401	0.548597
GPSGPAG	541.2496	0.728095	EASGPINFT	934.4396	0.546921
APGKGIL	654.4064	0.679452	PGLSGAPG	654.3337	0.542914
SGPAGPA	555.2653	0.667579	DASLPGNYG	892.3926	0.539879
PGPSGPA	581.2809	0.660942	GERGAPGIGGP	966.4882	0.539457
AGAPGPSGP	709.3395	0.641999	GEPGPAG	583.2601	0.521885
AFPPDVA	715.3541	0.641548	GPETGPRGAPGPA	1061.5254	0.519873
AGLYPGA	675.3591	0.640776	GESGNPGAPG	841.3566	0.519609
EASGPINF	833.3919	0.629953	VAPGKGIL	753.4749	0.511749
GNHAAIIKPL	1033.592	0.625024	AGAGPGA	541.2496	0.510304
APGAPGA	539.2703	0.619753	GGVPGGAPLAV	893.497	0.505766

**Table 2:** Active peptide antioxidant activity and toxicity prediction.

Peptide Sequence	Peptide Length	Antioxidant Activity Score	Toxicity
AGLYPGA	7	0.54491031	no
GEPGPAG	7	0.50003815	no
LPGGGPVL	8	0.53823709	no
APVAPGYGGG	10	0.53759086	no
AAAPAPAPAPAPAPA	15	0.53961134	no
AEPAPAPAPAPEPAPAPA	18	0.54290658	no
AEPAPAPAPAPEPAPAPAAPA	21	0.53858268	no

## Data Availability

The data presented in this study are available in this article.
